# Integrating 3D Digital Technology Advancements in the Fabrication of Orthodontic Aligner Attachments: An In Vitro Study

**DOI:** 10.3390/jcm14145093

**Published:** 2025-07-17

**Authors:** Riham Nagib, Andrei Chircu, Camelia Szuhanek

**Affiliations:** 1 Department of Orthodontics 1, Orthodontics Research Center ‘ORTHO CENTER’, “Victor Babeş” University of Medicine and Pharmacy Timişoara, Eftimie Murgu Sq. 2, 300041 Timişoara, Romania; 2 Private Dental Tehnician Laboratory, Horia Street 3, 300342 Timişoara, Romania

**Keywords:** aligner attachments, three-dimensional printing, digital orthodontics, intraoral scanning

## Abstract

**Background/Objectives**: The introduction of composite attachments has greatly improved orthodontic aligner therapy, through better force delivery, more predictable movements, and enhanced retention. This in vitro study aims to present and investigate an innovative digital protocol for aligner attachment fabrication incorporating the latest 3D technology used in dentistry. **Methods**: A virtual attachment measuring 2.5 × 2 × 2 mm was designed using computer-aided design (CAD) software (Meshmixer, Autodesk Inc., San Francisco, CA, USA) and exported as an individual STL file. The attachments were fabricated using a digital light processing (DLP) 3D printer (model: Elegoo 4 DLP, Shenzhen, China) and a dental-grade biocompatible resin. A custom 3D-printed placement guide was used to ensure precise positioning of the attachments on the printed maxillary dental models. A flowable resin was applied to secure the attachments in place. Following attachment placement, the models were scanned using a laboratory desktop scanner (Optical 3D Smart Big, Open Technologies, Milano, Italy) and three intraoral scanners: iTero Element (Align Technology, Tempe, AZ, USA), Aoral 2, and Aoral 3 (Shining 3D, Hangzhou, China). **Results**: Upon comparison, the scans revealed that the iTero Element exhibited the highest precision, particularly in the attachment, with an RMSE of 0.022 mm and 95.04% of measurements falling within a ±100 µm tolerance. The Aoral 2 scanner showed greater variability, with the highest RMSE (0.041 mm) in the incisor area and wider deviation margins. Despite this, all scanners produced results within clinically acceptable limits. **Conclusions**: In the future, custom attachments made by 3D printing could be a valid alternative to the traditional composite attachments when it comes to improving aligner attachment production. While these preliminary findings support the potential applicability of such workflows, further in vivo research is necessary to confirm clinical usability.

## 1. Introduction

The field of orthodontics has undergone significant digital transformation in recent years. Among the most impactful advancements is the development of removable clear aligners, which have gained widespread popularity for their esthetic appeal, patient comfort, and enhanced oral hygiene compared to traditional fixed appliances [1,2]. These aligners are fabricated from medical-grade thermoplastic materials and worn in sequential stages to guide teeth progressively toward their planned positions, as determined by digital treatment simulations [3].

However, clear aligners face biomechanical limitations, particularly in achieving complex tooth movements such as extrusion, rotation, bodily translation, and root torque [4,5]. These challenges primarily arise due to insufficient mechanical engagement between the aligner and the tooth surface. To enhance force application, composite resin attachments have become standard auxiliaries in aligner therapy [2,3]. These structures are bonded to the enamel and enhance aligner retention and precision in force application [6]. Typically, attachments are digitally designed using computer aided design software and transferred using vacuum-formed or silicone guides [7]. Shape accuracy and the consistency of attachment placement are influenced by several factors, including the template material [8,9], composite resin properties [10], and curing technique [11].

Recent advances in additive manufacturing, particularly digital light processing 3D printing, offer a promising alternative by enabling the production of highly precise, custom-designed attachments and transfer guides that match individual tooth morphology [12,13]. These 3D-printed components can reduce operator variability, improve reproducibility, and support clinical precision when fabricated from biocompatible and rigid materials [14]. Parallel to these developments, digital intraoral scanning has replaced conventional impression methods, offering increased patient comfort, faster digital workflow integration, and the generation of high-resolution models [15,16,17]. However, with a growing number of intraoral scanners on the market, clinicians use devices with varying accuracy, performance in capturing fine surface details and clinical integration potential [18,19]. Despite this, few studies have evaluated how well different intraoral scanners capture the geometry of small, detailed auxiliaries, which are critical for aligner effectiveness [20].

To address this gap, the present in vitro study is among the first to introduce an innovative digital protocol for fabricating and transferring 3D-printed orthodontic attachments using custom polymeric guides. It further assesses the ability of three different intraoral scanners to accurately capture the geometry of these attachments in comparison to a laboratory scanner. The null hypothesis is that there is no significant difference in the accuracy of capturing attachment morphology among the intraoral scanners models within the scope of routine intraoral scanning procedures.

## 2. Materials and Methods

### 2.1. Attachment Design and Fabrication Protocol

The orthodontic aligner attachments model was digitally designed using specialized computer-aided design (CAD) software and then fabricated using a three-dimensional (3D) printer with a biocompatible, dental-grade resin suitable for clinical applications.

#### 2.1.1. CAD

**Figure 1:**
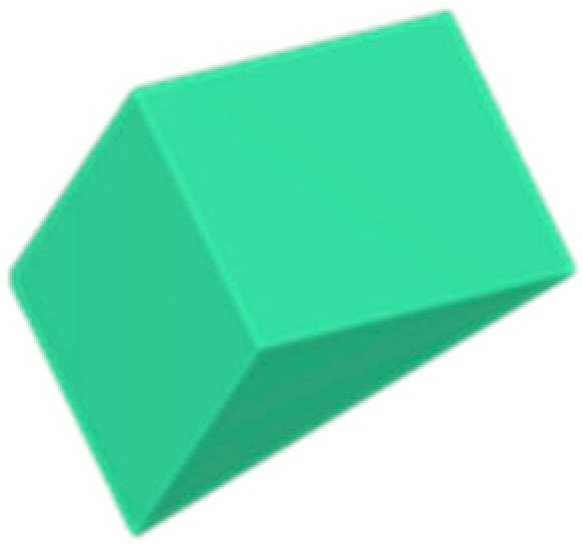
STL model of the attachments custom-designed for the current study.

A virtual attachment was custom-designed using computer-aided design (CAD) software (Meshmixer, Autodesk Inc., San Francisco, CA, USA). The pyramid shaped model presented the following dimensions: 2.5 mm in the cervical-incisal margin dimension, while mesio-distally the dimension was 2 mm. The vestibulo-lingual dimension measured 2 mm at the highest point. The virtual model was saved as individual STL files (Figure 1).

#### 2.1.2. Three-Dimensional Printing

The STL files were imported into a slicing software (CHITUBOX Basic V2, Shenzhen, China) to prepare the models for three-dimensional printing. The finalized printing project was transferred to the 3D printer (Elegoo 4K DLP, Shenzhen, China), and fabrication was performed using 3D printable resin (NextDent C&B Micro-Filled Hybrid, Vertex-Dental, Soesterberg, The Netherlands), a biocompatible material suitable for dental applications. The printed components were rinsed twice in 96% alcohol using an ultrasonic bath to eliminate excess resin material. After cleaning, they were thoroughly dried and subjected to final polymerization in a UV light-curing unit. Once cured, all support structures were carefully removed [21].

### 2.2. Attachment Bonding and Scanning Protocol

#### 2.2.1. Bonding Procedure

An intraoral scan was randomly selected from the patient database of a private clinic in Timisoara, Romania. The inclusion criteria were as follows: fully erupted permanent central upper incisors. The exclusion criteria were based on the following conditions: scanning defects or caries and restorations in the upper incisor area.

**Figure 2:**
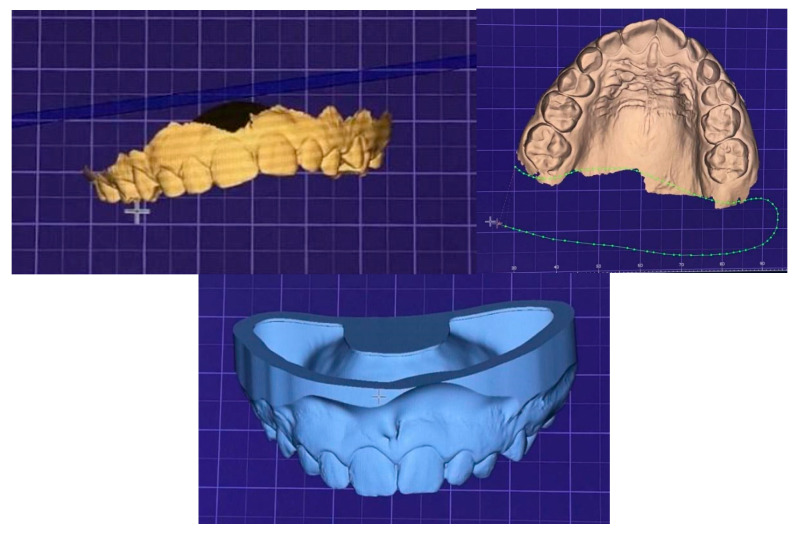
Patient intraoral scan imported in Exocad (DentalCAD 3.1 Rijeka, exocad GmbH, Darmstadt, Germany) software and the marginal adaptation process.

After selection, the STL file was imported into Exocad (exocad GmbH, Darmstadt, Germany), where the marginal adaptation of the scan was performed (Figure 2). The next step involved preparing the models for printing using the PreForm 3.42.0 (FormLabs Inc., Somerville, MA, USA) software. The models were printed using the Formlabs 3 (Formlabs Inc., USA) 3D printer, with Grey V4 resin from the same manufacturer.

The bonding procedure for the 3D-printed attachments was conducted with individualized bonding guides designed with the aid of computer aided design software and printed using the 3D printer (Elegoo 4 DLP, Shenzen, China) to ensure accurate and reproducible positioning on the tooth surface. The guides included tooth surfaces (central and lateral incisors—both vestibular and palatal areas) and were extended on the palatal mucosa for precise and stable positioning.

**Figure 3:**
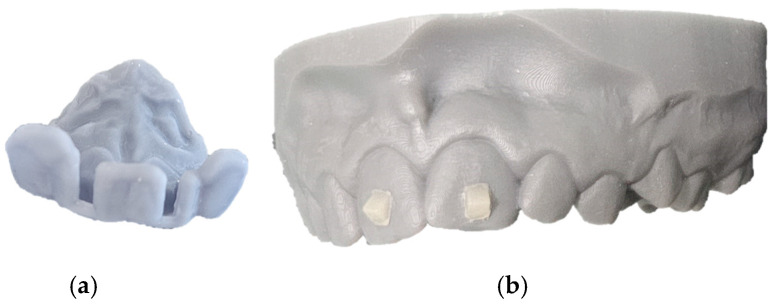
(**a**) Personalized bonding guides for reproducible and precise bonding position of the 3D printed attachments on the tooth surface; (**b**) 3D printed attachments bonded on the dental model upper central incisor surface.

The custom 3D printed attachments were inserted into the positioning guides and a flow composite (Filtek™ Supreme XT Flowable Restorative, 3M ESPE, Maplewood, MN, USA) was applied for bonding. The positioning guides were applied to the dental model and cured with a UV light-curing lamp (Valo, Ultradent, South Jordan, UT, USA) (Figure 3).

#### 2.2.2. Scanning

The printed models were scanned using a laboratory model scanner (Optical 3D Smart Big, Open Technologies, Cassano Magnago, Italy) and three types of intraoral scanners: iTero Element, Aoral 2, and Aoral 3. These scanners were selected to represent a range of scanning devices used in clinical practice.

All scans were performed by a single trained operator using a standardized scanning protocol. The process began at the occlusal surface of the molars, proceeded in a continuous sweep across the central incisors, and extended to the opposing molars. This was followed by systematic scanning of the buccal and palatal surfaces, as well as the palate. Throughout, the dental arch model remained in a fixed position to prevent any movement or distortion during the scanning session.

To ensure consistency and minimize bias, all scanning procedures were performed using a uniform technique. Scanning was performed by a single operator that maintained consistent scanning distances and movements based on the predefined protocols for each type of scanner. To control the operator-related variability, each scanner was used to perform ten consecutive scans following the same standardized sequence. This ensured comprehensive and repeatable scanning of the dental models. From the ten scans acquired per intraoral scanner, one scan was randomly selected for analysis. The random selection was performed to eliminate potential selection bias and to reflect conditions encountered in routine clinical workflows; typically only a single scan is acquired and used in treatment planning.

**Figure 4:**
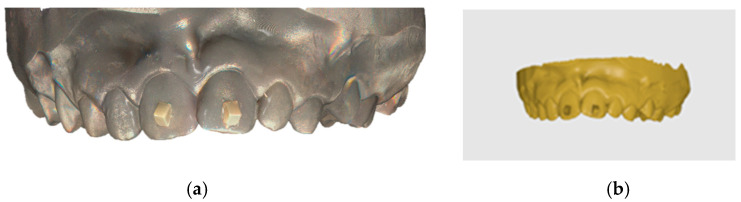
Scanned data: (**a**) Intraoral scanner result; (**b**) STL file uploaded in Medit Link—Medit Design v.3.4.3 (Seoul, Republic of Korea) software.

Environmental conditions (lighting, temperature, humidity) were rigorously monitored to eliminate potential sources of variation in scan accuracy and quality. All scans were conducted under identical conditions. The resulting scans were saved as STL files. For the analysis of scanned data, a standardized software program, Medit Link—Medit Design v.3.4.3 (Seoul, Republic of Korea), was chosen (Figure 4).

For each of the three types of scanners, the scanned models were superimposed using the ‘Alignment Mode tool’ in Medit Link—Medit Design v.3.4.3 (Seoul, Republic of Korea) software, allowing the independent alignment of each scan with the laboratory model scanner scan, used as a reference model. The ‘Align target data separately’ option was used to optimize alignment accuracy, supplemented by automatic alignment features to minimize manual errors. Measurement tools and software parameters were routinely calibrated to ensure consistent accuracy and reliability throughout the analysis.

Deviation analysis was conducted to quantify discrepancies between the scanned objects and the reference model. The software’s ‘Deviation Display Mode’ generated detailed color-coded deviation maps and tabulated reports, presenting dimensional differences and error margins in millimeters (mm). The ‘Curvature Display Mode,’ a feature of dental scanning software, provided the visualization of the curvature of surfaces, highlighting concave, convex, and flat areas using color gradients. Three areas for each type of scanner were taken into consideration separately and obtained by cutting out the rest of the scan data, in order to assess accuracy variations between different areas of the scanned models: total arch, upper incisors (central and lateral), and attachments. The parameters taken into consideration are listed in Table 1 and the data set was statistically analyzed.

## 3. Results

**Figure 5:**
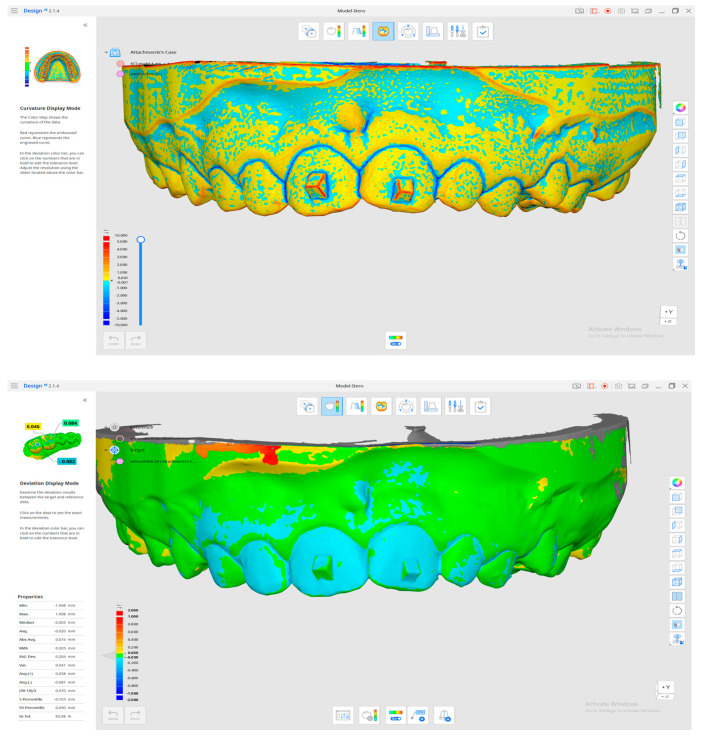
View from the Medit Link—Medit Design v.3.4.3 (Seoul, Republic of Korea) software: “curvature display mode” and “deviation display mode” superimpositions.

All intraoral scanning systems (iTero Element, Align Technology, USA; Aoral 2 and Aoral 3, Shining 3D, China) evaluated in this study demonstrated both positive and negative dimensional deviations when compared to the reference control model (Figure 5). These deviations indicate the presence of minor distortions or inconsistencies in the scanned data, which were not limited to a single system but observed across all tested devices. Despite the implementation of a uniform scanning protocol and consistent scanning techniques, designed to minimize operator-induced variability, noticeable differences emerged between the initial virtual attachment model (no curved surfaces) and the scanned attachments in the ‘Curvature Display Mode’.

In the attachments region, Aoral 2 and Aoral 3 scanners show nearly identical RMSE and standard deviation values (0.028 mm, respectively, 0.027 mm), while iTero Element scanner exhibits the lowest values (0.022 mm), indicating greater precision and consistency. Trueness also improves progressively from Aoral 2 (0.032 mm) to Aoral 3 (0.028 mm) to iTero (0.023 mm), confirming iTero Element’s superior ability to reproduce accurate attachment geometry (Table 2).

**Figure 6:**
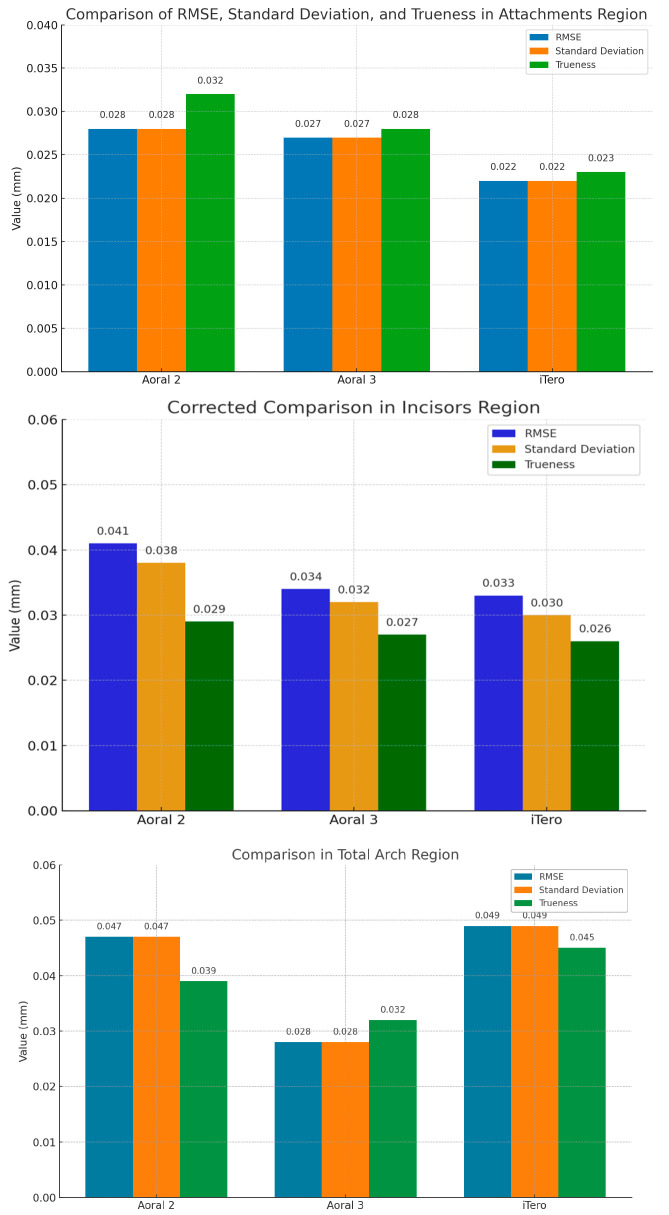
Visual comparison of Aoral 2, Aoral 3, and iTero scanners results—RMSE, standard deviation, and trueness in the attachments, incisors, and total arch regions.

In the incisors region, the Aoral 2 scanner presents the highest RMSE (0.041 mm) and trueness (0.029 mm), reflecting greater deviation. Aoral 3 scanners offer improved precision, but iTero Element demonstrates the highest consistency with the lowest standard deviation (0.030 mm). For the total arch, Aoral 3 outperforms the others in overall accuracy, with the lowest RMSE (0.028 mm) and trueness (0.032 mm). Although the Aoral 2 and iTero Element scanners have similar standard deviations (0.047 mm and 0.049 mm), the iTero Element shows the highest RMSE (0.049 mm), indicating occasional larger deviations (Figure 6).

In full-arch scans, Aoral 2 exhibited the most extreme minimum deviation (−1.941 mm), followed by iTero (−1.777 mm), indicating occasional significant underestimation. Aoral 3 showed the smallest minimum deviation (−0.106 mm), suggesting better control over extreme errors. Maximum deviations mirrored this trend, with iTero having the highest value (0.472 mm) and Aoral 3 the lowest (0.098 mm), highlighting its reduced overestimation tendency. Aoral 2 sample showed a median value of −0.011 mm in the incisor region, suggesting a mild underestimation trend. For full arch scans, Aoral 3 showed the narrowest deviation range (−0.042 mm to 0.048 mm), with iTero close behind (−0.070 mm to 0.054 mm). Aoral 2 showed a broader spread with values ranging between −0.060 mm to 0.046 mm, indicating a higher likelihood of deviations beyond the clinical threshold. In the incisor region, iTero provided the most stable results (−0.042 mm to 0.022 mm), followed by Aoral 3 and Aoral 2, which both stayed within tolerance limits but with greater variability in the latter. In the critical attachment region, iTero again led (−0.034 mm to 0.035 mm), with Aoral 3 (−0.050 mm to 0.040 mm) and Aoral 2 (−0.042 mm to 0.048 mm) slightly more variable.

Overall, the analysis of the RMSE, standard deviation, and trueness in the attachments, incisors, and total arch regions revealed that iTero Element leads in precision in critical areas such as attachments and incisors, while Aoral 3 provides consistently accurate full-arch scanning, and Aoral 2 shows more variability and lower reliability across all regions. As mentioned, these discrepancies occurred regardless of the specific scanner used, suggesting that factors inherent to the scanning technology or the geometry of the scanned region may contribute to variability in outcomes.

The anterior region of the dental arch consistently exhibited higher accuracy and reproducibility in scanned data compared to the posterior region. A comparison between minimum, maximum, mean, and median deviations revealed clear differences in scanner performance. The results may be attributed to the relatively less complex morphology and better accessibility of the anterior area, which facilitates improved image capturing and alignment. Across incisor and attachment regions, deviations were generally more moderate. Aoral 2 had the widest range of deviations, while Aoral 3 demonstrated the most stable output. Mean deviations for all scanners ranged from −0.014 mm to 0.000 mm, with median values near zero, indicating a balanced error distribution.

## 4. Discussion

The first part of the study introduces a protocol that uses prefabricated 3D-printed orthodontic attachments, which are bonded directly to the enamel surface using customized 3D-printed guides, similar to the bonding process for fixed orthodontic brackets. This approach enhances precision, reproducibility, and efficiency [22] compared to conventional methods that rely on vacuum-formed or silicone transfer templates. Traditional techniques often lack standardized composite volume and can lead to issues such as resin overflow and poor structure, which can compromise aligner fit [23,24]. Additionally, the accuracy of attachment placement in these methods is only confirmed after light curing, making real-time corrections difficult and increasing the risk of undesired clinical outcomes [3,10].

In contrast, the 3D-printed attachments are virtually designed and fabricated using high-resolution printers and biocompatible resins, ensuring consistent shape and size. Clinicians are thus able to visually verify accuracy prior to bonding, reducing post-curing adjustments [12,13]. Clinically, only a thin adhesive layer (approximately 200 µm) is applied, requiring minimal light exposure [25], which decreases chair time and improves curing uniformity.

Studies have shown that the degree of polymerization is reduced when composite is cured beneath the aligner, due to light diffusion and attenuation, potentially affecting the bond strength and long-term durability of the attachments [26]. By shifting the curing step to the lab phase, the new method optimizes bond strength and attachment durability. Because aligners are fabricated to match the geometry and position of these attachments, any bonding inaccuracies can result in unintended tooth movement or treatment inefficiencies [4,5,9]. The proposed protocol reduces such risks by increasing control over attachment placement, offering a more predictable and clinically effective solution for aligner therapy. Moreover, these attachments are fully polymerized during the printing post-processing phase, eliminating the need for extensive intraoral curing [27,28].

As digital workflows continue to advance in orthodontics, the integration of 3D-printed attachments with accurate intraoral scanning represents a significant step forward in enhancing the predictability and quality of aligner-based treatments. One of the main goals of this study was to evaluate the reliability of data transfers between clinics and dental laboratories, particularly when using digital tools [22,29,30].

The second part of the study investigates deviations between 3D attachments scanned with intraoral scanners and a reference model—the model scanned with the laboratory model. The literature in the field states that model and extraoral scanning have greater accuracy than intraoral scanning [31,32]. The findings confirm that combining prefabricated 3D-printed attachments with precise intraoral scanning offers a reliable, consistent, and clinically efficient protocol and are in line with the current literature [15,30]. However, an area that remains underexplored is directly comparing how different intraoral scanners capture small, detailed 3D-printed attachments. Both Aoral 3 and iTero performed within clinically acceptable tolerance limits (±0.05 mm), making them suitable for high-precision applications, while Aoral 2, although within tolerance, showed greater variability and may benefit from further refinement in high-detail tasks. Consistent with the prior literature, our results fall within the accuracy range demonstrated in other studies [22,29,32,33]. An in vivo study has shown median trueness of ~0.015 mm and full-arch precision differences of only ~0.01 mm—differences deemed clinically insignificant yet informative when selecting scanners for high-detail tasks such as attachment capturing [34]. These findings reinforce the significance of scanning protocol in ensuring reliable intraoral digitization, especially in workflows that depend heavily on precise morphological reproduction.

From a practical standpoint, the cost–benefit balance of incorporating 3D-printed attachments in routine orthodontic workflows warrant consideration. This approach offers potential advantages in terms of reduced chair time, fewer errors, improved bond quality, and better integration with digital aligner systems, factors that can ultimately offset upfront costs, especially in high-volume or digitally integrated clinics [14]. However, the initial investment in CAD software, high-resolution printers, and dental-grade resins may still be a barrier for some practices [34].

Future in vivo phases of this research will incorporate larger sample sizes and focus on quantitative comparisons to further validate the protocol. While we acknowledge the limitations of the current in vitro design—particularly regarding statistical generalizability and the reproducibility of attachment placement across diverse tooth morphologies—this controlled approach was intentionally selected to simulate key aspects of clinical practice. By establishing a standardized and repeatable protocol under realistic conditions, this study’s contribution may inspire further research, ultimately supporting the integration of 3D-printed attachments into routine clinical workflows.

## 5. Conclusions

3D-printed orthodontic attachments provide a valid alternative to the traditional composite attachments when it comes to improving aligner attachment production. The findings of the present study confirm that these types of attachments can be accurately captured by intraoral scanners in in vitro conditions. In the future, well-designed clinical studies should incorporate multiple operators, varied anatomical regions, and more complex in vivo conditions to provide a comprehensive assessment of the applicability of the novel protocols and support their effective integration into fully digital clear aligner workflows.

## Figures and Tables

**Table 1:** Overview of parameters generated by the analysis software.

Parameters	Definition	Purpose
**Mean**	Average of all deviation values	Indicates the typical deviation per scanner
**Median**	Middle value when data are ordered	Reflects central tendency, less affected by outliers
**Mean Absolute Deviation (MAD)**	Average of absolute deviations from the mean	Measures consistency: lower MAD implies higher precision
**Root Mean Square Error (RMSE)**	Square root of average squared deviations from the mean	Emphasizes larger errors; indicates overall accuracy
**Standard Deviation (SD)**	Dispersion of values around the mean	Shows how tightly deviations cluster; lower SD equals more consistent results
**Variance**	Square of the standard deviation	Quantifies the overall variability within the data set
**Trueness [(P90 − P10)/2]**	Half the range between the 90th and 10th percentile values	Indicates accuracy by assessing the spread of central 80% of data
**Tolerance**	Percentage of deviations within a predefined acceptable range	Measures how often scanner deviations fall within clinically acceptable limits

**Table 2:** Scan data deviations for each type of scanner compared to the reference model in the three areas considered in the study: total arch; upper incisors and attachments.

Parameters	Total			Incisors			Attachments		
	Aoral 2	Aoral 3	iTero	Aoral 2	Aoral 3	iTero	Aoral 2	Aoral 3	iTero
Min. (mm)	−1.941	−0.106	−1.777	−0.325	−0.273	−0.242	−0.106	−0.127	−0.110
Max. (mm)	0.463	0.098	0.472	0.362	0.172	0.239	0.098	0.128	0.089
Median (mm)	−0.005	−0.002	0.007	−0.011	−0.007	−0.010	−0.002	−0.005	−0.000
Avg. (mm)	−0.004	0.001	−0.006	−0.014	−0.011	−0.013	0.001	−0.003	−0.000
Abs Avg. (mm)	0.029	0.021	0.032	0.024	0.020	0.021	0.021	0.019	0.016
RMSE (mm)	0.047	0.028	0.049	0.041	0.034	0.033	0.028	0.027	0.022
Std. Dev. (mm)	0.047	0.028	0.049	0.038	0.032	0.030	0.028	0.027	0.022
Variance (mm)	0.002	0.001	0.002	0.001	0.001	0.001	0.001	0.001	0.000
Avg. (+) (mm)	0.028	0.023	0.032	0.017	0.013	0.015	0.023	0.021	0.015
Avg. (−) (mm)	−0.029	−0.018	−0.032	−0.026	−0.023	−0.023	−0.018	−0.019	−0.016
Trueness (mm)	0.039	0.032	0.045	0.029	0.027	0.026	0.032	0.028	0.023
5 Percentile (mm)	−0.060	−0.042	−0.070	−0.066	−0.059	−0.064	−0.042	−0.050	−0.034
95 Percentile (mm)	0.046	0.048	0.054	0.024	0.025	0.022	0.048	0.040	0.035
In Tol. (%)	87.50	91.66	83.34	90.95	92.48	92.33	91.66	92.02	95.04

## Data Availability

The data presented in this study is available on request from the corresponding author.

## References

[1] Barrera-Chaparro J.P., Plaza-Ruíz S.P., Parra K.L., Quintero M., Velasco M.D.P., Molinares M.C., Álvarez C. (2023). Orthodontic treatment need, the types of brackets and the oral health-related quality of life. Dent. Med. Probl..

[2] Shashidhar K., Kanwal B., Kuttappa M., Nayak U.K., Shetty A., Mathew K. (2022). Clear aligners: Where are we today? A narrative review. J. Int. Oral Health.

[3] Jedliński M., Mazur M., Greco M., Belfus J., Grocholewicz K., Janiszewska-Olszowska J. (2023). Attachments for the orthodontic aligner treatment—State of the art: A comprehensive systematic review. Int. J. Environ. Res. Public Health.

[4] Ho C.T., Huang Y.T., Chao C.W., Huang T.H., Kao C.T. (2021). Effects of different aligner materials and attachments on orthodontic behavior. J. Dent. Sci..

[5] Chen W., Qian L., Qian Y., Zhang Z., Wen X. (2021). Comparative study of three composite materials in bonding attachments for clear aligners. Orthod. Craniofac. Res..

[6] Kuncio D., Maganzini A., Shelton C., Freeman K. (2014). In vitro evaluation of the retention of attachments used for aligner therapy. J. Clin. Orthod..

[7] Rossini G., Parrini S., Castroflorio T., Deregibus A., Debernardi C.L. (2015). Efficacy of clear aligners in controlling orthodontic tooth movement: A systematic review. Angle Orthod..

[8] Dasy H., Dasy A., Asatrian G., Rózsa N., Lee H.-F., Kwak J.H. (2015). Effects of variable attachment shapes and aligner material on aligner retention. Angle Orthod..

[9] Weckmann J., Scharf S., Graf I., Schwarze J., Keilig L., Bourauel C., Braumann B. (2020). Influence of attachment bonding protocol on precision of the attachment in aligner treatments. J. Orofac. Orthop..

[10] D’Antò V., Muraglie S., Castellano B., Candida E., Sfondrini M.F., Scribante A., Grippaudo C. (2019). Influence of dental composite viscosity in attachment reproduction: An experimental in vitro study. Materials.

[11] Yaosen C., Mohamed A.M., Jinbo W., Ziwei Z., Al-Balaa M., Yan Y., Grassia V. (2021). Factors of composite attachment loss in orthodontic patients during orthodontic clear aligner therapy: A prospective study. Biomed. Res. Int..

[12] Nagib R., Farkas A.Z., Szuhanek C. (2024). Finite element analysis of the mechanical behavior of 3D-printed orthodontic attachments used in aligner treatment. Sci. Rep..

[13] Bellocchio A.M., Ciancio E., Ciraolo L., Barbera S., Nucera R. (2024). Three-dimensional printed attachments: Analysis of reproduction accuracy compared to traditional attachments. Appl. Sci..

[14] Paľovčík M., Tomášik J., Zsoldos M., Thurzo A. (2025). 3D-printed accessories and auxiliaries in orthodontic treatment. Appl. Sci..

[15] Christopoulou I., Kaklamanos E.G., Makrygiannakis M.A., Bitsanis I., Perlea P., Tsolakis A.I. (2022). Intraoral scanners in orthodontics: A critical review. Int. J. Environ. Res. Public Health.

[16] Pesce P., Nicolini P., Caponio V.C.A., Zecca P.A., Canullo L., Isola G., Baldi D., De Angelis N., Menini M. (2025). Accuracy of full-arch intraoral scans versus conventional impression: A systematic review with a meta-analysis and a proposal to standardise the analysis of the accuracy. J. Clin. Med..

[17] Nedelcu R., Olsson P., Nystrom I., Thor A. (2018). Finish line distinctness and accuracy in 7 intraoral scanners versus conventional impression: An in vitro descriptive comparison. BMC Oral Health.

[18] Ciocan L.T., Vasilescu V.G., Răuță S.-A., Pantea M., Pițuru S.-M., Imre M. (2024). Comparative analysis of four different intraoral scanners: An in vitro study. Diagnostics.

[19] Farah R.I., Alresheedi B., Alazmi S., Ali S.N.A. (2025). Evaluating the impact of scan body angulation and geometric attachments on the accuracy of complete-arch digital implant impressions: A comparison of two intraoral scanners. J. Prosthodont..

[20] Selvaraj A., Dinesh S.P.S., Sivakumar A., Arvind T.R.P., Albar D.H., Alshehri A., Awadh W., Alzahrani K.J., Halawani I.F., Alshammeri S. (2023). Evaluation of scanning accuracy for two commercially available intraoral scanners in reproducing orthodontic bracket dimensions. Eur. Rev. Med. Pharmacol. Sci..

[21] Nagib R., Szuhanek C., Moldoveanu B., Negrutiu M.L., Sinescu C., Brad S. (2017). Custom-designed orthodontic attachment manufactured using a biocompatible 3D printing material. Mater. Plast..

[22] Mehl A., Reich S., Beuer F., Güth J.F. (2021). Accuracy, trueness, and precision—A guideline for the evaluation of these basic values in digital dentistry. Int. J. Comput. Dent..

[23] Koenig N., Choi J.-Y., McCray J., Hayes A., Schneider P., Kim K.B. (2022). Comparison of dimensional accuracy between direct-printed and thermoformed aligners. Korean J. Orthod..

[24] Mantovani E., Castroflorio E., Rossini G., Garino F., Cugliari G., Deregibus A., Castroflorio T. (2019). Scanning electron microscopy analysis of aligner fitting on anchorage attachments. J. Orofac. Orthop..

[25] Filtek Supreme XT Flowable Restorative 3M Product Document. https://multimedia.3m.com/mws/media/598213O/filtek-supreme-xt-flow-tpp.pdf.

[26] Lasance S.J., Koletsi D., Eliades G., Eliades T. (2024). Degree of cure of orthodontic composite attachments underneath aligners. Eur. J. Oral Sci..

[27] Can E., Panayi N., Polychronis G., Papageorgiou S.N., Zinelis S., Eliades G., Eliades T. (2022). In-house 3D-printed aligners: Effect of in vivo ageing on mechanical properties. Eur. J. Orthod..

[28] von Glasenapp J., Hofmann E., Süpple J., Jost-Brinkmann P.-G., Koch P.J. (2022). Comparison of two 3D-printed indirect bonding (IDB) tray design versions and their influence on the transfer accuracy. J. Clin. Med..

[29] Amornvit P., Rokaya D., Sanohkan S. (2021). Comparison of accuracy of current ten intraoral scanners. Biomed. Res. Int..

[30] Lione R., De Razza F.C., Gazzani F., Lugli L., Cozza P., Pavoni C. (2024). Accuracy, time, and comfort of different intraoral scanners: An in vivo comparison study. Appl. Sci..

[31] San José V., Bellot-Arcis C., Tarazona B., Zamora N., O Lagravere M., Paredes-Gallardo V. (2017). Dental measurements and Bolton index reliability and accuracy obtained from 2D digital, 3D segmented CBCT, and 3D intraoral laser scanner. J. Clin. Exp. Dent..

[32] Flügge T.V., Schlager S., Nelson K., Nahles S., Metzger M.C. (2013). Precision of intraoral digital dental impressions with iTero and extraoral digitization with the iTero and a model scanner. Am. J. Orthod. Dentofac. Orthop..

[33] Winkler J., Gkantidis N. (2020). Trueness and precision of intraoral scanners in the maxillary dental arch: An in vivo analysis. Sci. Rep..

[34] Ergül T., Güleç A., Göymen M. (2023). The use of 3D printers in orthodontics—A narrative review. Turk. J. Orthod..

